# Disulfiram/Copper Combination as a Potential Therapeutic Approach for Hepatocellular Carcinoma: Targeting the ATF3-Mitochondrial Cell Death Pathway

**DOI:** 10.7150/jca.113442

**Published:** 2026-01-01

**Authors:** Jing Cao, Jing Deng, Xinhua Li, Yaqiong Chen, Jialei Wang, Yutian Chong, Jiao Gong, Bingliang Lin

**Affiliations:** 1 Department of Infectious Diseases, Key Laboratory of Liver Disease of Guangdong Province, Third Affiliated Hospital of Sun Yat-sen University, Guangzhou, 510630, China.; 2 Department of Laboratory Medicine, Third Affiliated Hospital of Sun Yat-sen University, Guangzhou, 510630, China.

**Keywords:** disulfiram, hepatocellular carcinoma, mitochondrial damage, apoptosis, ATF3

## Abstract

Hepatocellular carcinoma (HCC) represents a major public health issue globally, necessitating the urgent development of new therapies. The therapeutic efficacy of disulfiram (DSF) and copper (Cu) in HCC was investigated in the present study, focusing on cytotoxicity, mitochondrial function, and apoptosis to clarify the mechanistic basis of this drug combination. Our findings revealed a significant, dose-dependent reduction in HCC cell viability with DSF/Cu treatment. Further investigation showed increased reactive oxygen species (ROS) levels, decreased adenosine triphosphate (ATP) production, and a decline in mitochondrial membrane potential (MMP). These events culminated in the activation of caspase-9 and caspase-3, key enzymes in the apoptotic pathway, leading to cell death. Mechanistically, DSF/Cu synergistically increased the expression of activating transcription factor 3 (ATF3), a known tumor suppressor, in HCC cells. *In vivo* studies using a mouse tumor model supported these findings, demonstrating significantly inhibited tumor growth in the DSF/Cu group compared with the control group. Overall, our study findings suggest that the DSF/Cu combination exhibits significant therapeutic potential against HCC by modulating the ATF3-dependent mitochondrial apoptosis pathway, a strategy that warrants further preclinical exploration.

## Introduction

Hepatocellular carcinoma (HCC) is the most common type of primary liver cancer, accounting for 75%-86% of cases. As a major global health concern, it imposes a substantial strain on healthcare systems worldwide 1. Despite advancements in therapeutic approaches, the prognosis for advanced-stage HCC remains suboptimal, necessitating the exploration of innovative and effective treatment strategies 2. Despite considerable progress in liver cancer diagnostics and therapeutics, surgical resection, liver transplantation, and radiofrequency ablation remain the mainstay of treatment for early-stage HCC 3. Due to the inherent difficulties in detecting early-stage disease, the majority of cases are diagnosed at advanced phases, where molecular targeted therapy and immunotherapy play critical roles. Nevertheless, due to challenges such as high drug resistance and frequent recurrence, the overall prognosis of this patient population remains poor 4. Therefore, identifying novel therapeutic strategies is crucial for improving clinical outcomes. Indeed, the high costs, failure rates, and long testing periods associated with developing new medicines emphasize the need for exploring alternative approaches. Repurposing existing drugs for anticancer purposes offers a faster and more cost-effective option 5. In recent years, this approach has gained significant momentum, with disulfiram (DSF), a U.S. Food and Drug Administration (FDA)-approved medication for alcohol use disorder, emerging as a potential candidate in cancer therapy 6. Furthermore, when combined with copper ions (Cu), DSF forms a complex known as the disulfiram/copper complex (DSF/Cu), demonstrating enhanced anticancer effects 7. This study explored the intricate mechanisms underlying the anticancer effects of DSF/Cu, specifically in the context of HCC. By examining the molecular pathways impacted by this unique combination, we sought to contribute to the understanding of its therapeutic potential and pave the way for novel and targeted interventions in the management of HCC.

Interestingly, activating transcription factor 3 (ATF3), a member of the ATF/cAMP-responsive element-binding protein (CREB) family, has been implicated in the response to various cellular stresses 8. Our study demonstrated that DSF/Cu could upregulate ATF3 in HCC, leading to mitochondrial damage, increased reactive oxygen species (ROS) production, and promotion of apoptosis in HCC cells. This highlights its significance in inhibiting HCC progression. Moreover, the efficacy of combining lenvatinib with DSF/Cu was investigated, providing crucial insights for the clinical use of DSF in HCC treatment.

## Materials and Methods

### Cell culture

HCC cell lines HepG2, SNU-449, Huh-7, and Hepa1-6 were purchased from the Cell Bank of Type Culture Collection (Shanghai, China). HepG2 and Huh-7 were cultured in Dulbecco's Modified Eagle Medium (DMEM) medium (C11995500BT, GIBCO, USA), while SNU-449 and Hepa1-6 were cultured in Roswell Park Memorial Institute-1640 (RPMI-1640) medium (C11875500BT, GIBCO, USA). These media were supplemented with 10% fetal bovine serum (FBS)(Gibco, Carlsbad, CA, USA) and 1% penicillin/streptomycin (Invitrogen) at 37 °C in a humidified air atmosphere containing 5% carbon dioxide. Both cell lines used in this study have been authenticated within the past year using short tandem repeat (STR) profiling.

### CCK-8 and TUNEL assays

HepG2 and SNU-449 cells were seeded at a density of 3,000 cells per well in 96-well plates. Following incubation, the cells were then exposed to various treatments: no treatment (control), DSF (different concentrations), Cu^2+^ (10μM, from MB2712-1, Meilunbio, China) 9, or a combination of both. After 24 hours, cell viability was measured using a Cell Counting Kit-8 (CCK-8) kit (APExBIO, K1018, Houston, USA) via absorbance measurement at 450 nm with a spectrophotometer. Three independent replicates were performed for each treatment group.

SNU-449 and HepG2 cells were seeded at different densities in 48-well plates (SNU-449: 3500 cells/well, HepG2: 4000 cells/well) and then treated with different treatments (control, DSF (200nM), Cu^2+^ (10 μM), or a combination of both) for 24 hours. Apoptosis in HepG2 cells was evaluated using the TUNEL Apoptosis Assay Kit (Green Fluorescence, C1086, Beyotime, China) according to the manufacturer's instructions. Images were captured using an inverted fluorescence microscope (Carl Zeiss, Jena, Germany). The proportion of TUNEL-positive cells (HepG2) was determined relative to DAPI-positive cells. The experiments were performed independently in triplicate.

### Mitochondrial membrane potential assay

To assess mitochondrial membrane potential, the JC-1 staining kit (C2003S, Beyotime, China) was employed. Following a 24-hour incubation with various treatments, cells were incubated with 10 μM JC-1 working solution for 30 minutes at 37°C in the dark. After washing with JC-1 Staining Buffer, the mitochondrial membrane potential (MMP) of fluorescently labeled cells was analyzed using flow cytometry. JC-1 monomers were detected using an excitation wavelength of 490 nm and an emission wavelength of 530 nm, while JC-1 aggregates were detected with an excitation wavelength of 525 nm and an emission wavelength of 590 nm.

### ROS and ATP assays

Intracellular ROS levels were measured using a 2',7'-dichlorofluorescein diacetate (DCFH-DA) probe (D6883, Sigma, USA). HepG2 and SNU-449 cells were seeded at a density of 2-3 × 10^5^ cells/well on 6-well culture plates and subjected to different treatment conditions for 24 hours. After washing with PBS, the cells were incubated with 10 µM DCFH-DA working solution at 37°C for 30 minutes. After 24 hours, intracellular ATP levels were measured using an ATP Assay Kit (S0027, Beyotime, China), in strict accordance with the manufacturer's protocol. Luminescence signals were measured and calculated using a Tecan Spark 10M multifunctional microplate reader (Tecan Group Ltd., Switzerland).

### Subcutaneous tumor xenograft model in mice

For the *in vivo* experiments, sixteen 5-week-old male NOD/ShiLtJGpt-*Prkdc^em26Cd52^Il2rg ^em26Cd22^* /Gpt mice were subcutaneously injected with 1 × 10^7^ HepG2 cells in the inguinal fold. After the tumor volume reached 100 mm³, the mice were randomly divided into three groups (n=4 per group) based on tumor size: control, DSF, and DSF/Cu. Mice in the DSF group received 60 mg/kg/day DSF (S1680, Selleck, Houston, USA) via intraperitoneal (i.p.) injection. Mice in the DSF/Cu group received DSF (60 mg/kg/day, i.p.) followed by Cu^2+^ (0.4 mg/kg/day, intragastric (i.g.) from MB2712-1, Meilunbio, China). All drug administrations continued for 3 weeks. Mice were monitored daily for clinical signs, including feeding behavior and activity levels. Tumor size and mouse weight were measured every two days. At the study endpoint, all mice were euthanized under anesthesia, and blood and tumors were collected for further analysis.

To further demonstrate the anti-tumor effect of DSF and explore the combined impact of lenvatinib and DSF, we obtained 5-week-old male NOD/ShiLtJGpt-*Prkdc^em26Cd52^Il2rg ^em26Cd22^* /Gpt mice (n = 35). To assess the anti-tumor effect of DSF, 1 × 10^6^ Hepa1-6 cells were subcutaneously injected into the inguinal fold of these mice. The experimental design comprised two distinct phases. In the first phase, we further validated the anti-tumor efficacy of DSF/Cu. Once tumor volumes reached the predetermined threshold, 15 tumor-bearing mice were randomly allocated into three groups (n=5 per group): control, DSF, and DSF/Cu combination. The second phase consisted of an independent experiment to investigate the potential synergistic effects of lenvatinib and DSF. For this purpose, Hepa1-6 cells were implanted subcutaneously into a supplementary cohort of twenty mice prior to treatment. After tumor establishment, the mice were allocated into four groups (n = 5 per group) based on tumor volume: vehicle control, DSF/Cu, Lenvatinib, and DSF/Cu plus Lenvatinib. Mice in the Lenvatinib group received 20 mg/kg/day Lenvatinib (S1164, Selleck, Houston, TX, USA) via intraperitoneal injection. Mice in the DSF/Cu+Lenvatinib group received DSF (60 mg/kg/day, i.p.) followed by Cu^2+^ (0.4 mg/kg/day, intragastric (i.g.) from MB2712-1, Meilunbio, China). All drug administrations continued for 3 weeks. Mice were monitored daily for clinical signs, including feeding behavior and activity levels. Tumor size and mouse weight were measured every two days. At the study endpoint, all mice were euthanized under anesthesia, and tumors were collected for further analysis.

### IHC and TUNEL assays

Immunohistochemistry (IHC) assays were performed for Ki-67 expression with an anti-Ki-67 antibody (GB111499, 1:400 dilution, Servicebio, Wuhan, China). TUNEL staining was performed using the TUNEL Apoptosis Assay Kit (Red Fluorescence) (C1089, Beyotime, China) according to the manufacturer's instructions. Images were captured using an upright fluorescence microscope (Carl Zeiss, Jena, Germany).

### Assessment of liver, kidney, and heart function

At the study endpoint, blood samples were collected from the mice, and serum was extracted. Serum levels of alanine aminotransferase (ALT), blood urea nitrogen (BUN), creatinine, and creatine kinase were measured to assess liver, kidney, and cardiac function, respectively. These analyses were performed at the Department of Laboratory Medicine, Third Affiliated Hospital of Sun Yat-sen University.

Detailed methodologies for bioinformatics analysis, functional enrichment, and subsequent experimental validation, including qPCR, western blotting, and Oil Red O staining, are provided in Supplementary Material 1.

### Statistical analysis

Statistical analysis was performed using GraphPad Prism software (GraphPad Software, San Diego, CA). Data were presented as means ± standard deviations (SD). A two-tailed Student's *t*-test was employed to compare two groups. Statistical significance was set at *p* < 0.05. Each experiment comprised at least three independent samples and was repeated at least three times to ensure robustness and reliability.

## Results

### Disulfiram and copper co-treatment inhibited HCC growth in a dose-dependent manner *in vitro*

**Figure 1:**
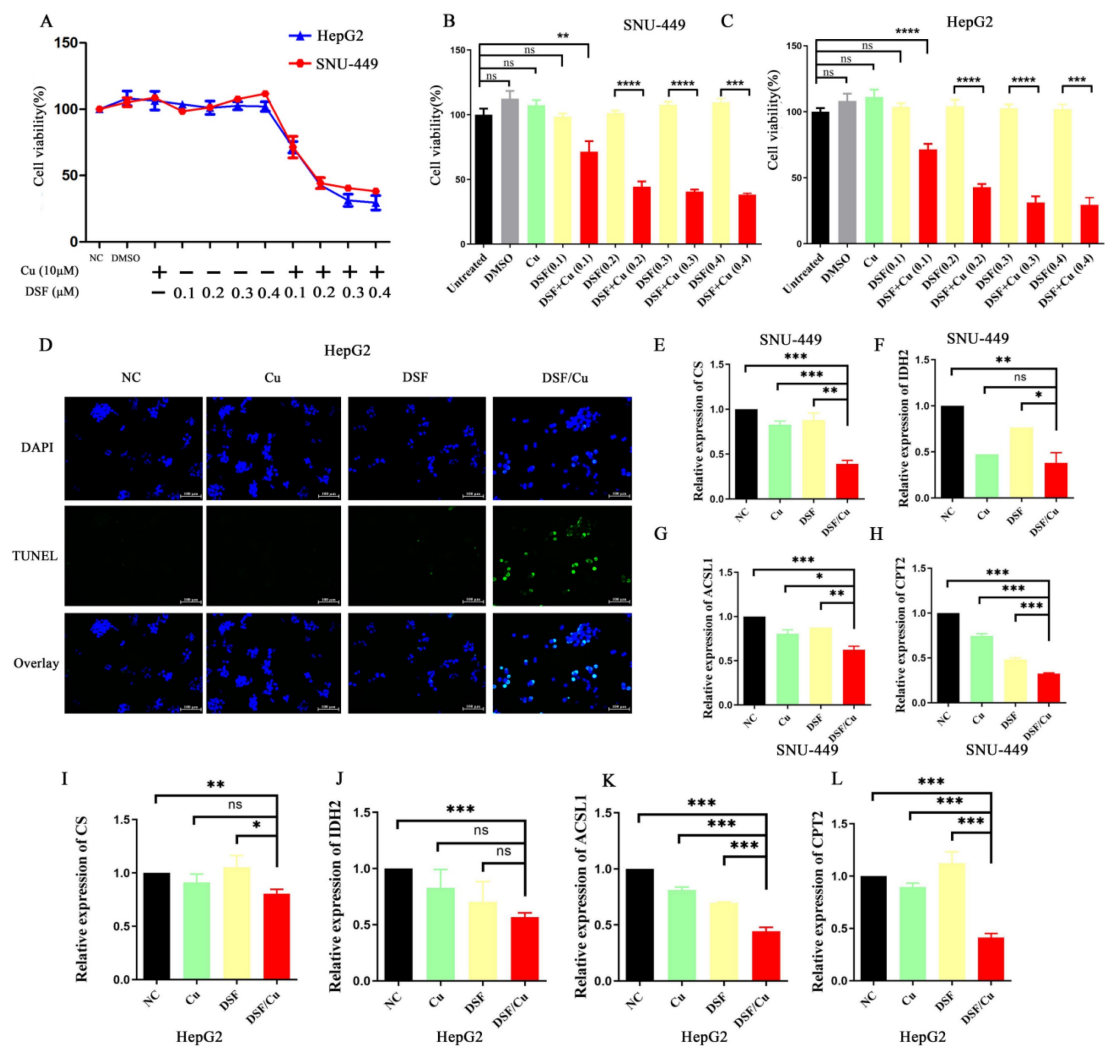
** Disulfiram combined with copper inhibited HCC cell proliferation, promoted apoptosis, and disrupted mitochondrial function.** A-C. Treatment of hepatocellular carcinoma cell lines (HepG2 and SNU-449) with disulfiram and copper ions for 24 hours showed concentration-dependent inhibition of cell viability by the combination. D. Combination treatment promoted apoptosis of hepatocellular carcinoma (HepG2) cells. E-L. Treatment with the combination downregulated the expression of CS, IDH2, ACSL1, and CPT2 in hepatocellular carcinoma cell lines, with data from the SNU-449 and HepG2 cell lines shown in panels E-H and I-L, respectively. *p < 0.05, **p < 0.01, ***p < 0.001. CS: Citrate Synthase, IDH2: Isocitrate Dehydrogenase 2, ACSL1: Acyl-CoA Synthetase Long Chain Family Member 1, and CPT2: Carnitine Palmitoyl Transferase 2.

To assess the antitumor efficacy of disulfiram or Cu^2+^ on HCC, we observed the morphological changes in SNU-449 and HepG2 cells treated with disulfiram, Cu^2+^, and their combination. Quantitative assessment of treatment efficacy was performed using CCK-8 viability assays. The DSF/Cu combination significantly decreased cell viability in both cell lines (**Figure 1A-C**). To further explore the underlying mechanisms, we conducted TUNEL assays. The results (**Figure 1D**) revealed that DSF/Cu induced apoptosis in HepG2 cells. Taken together, these findings suggest that DSF/Cu could effectively inhibit cell proliferation and induce apoptosis *in vitro*.

### DSF/Cu inhibited mitochondrial function and mediated cell apoptosis

Apoptosis, a form of programmed cell death triggered by specific physiological or pathological conditions, is tightly regulated by mitochondria. These organelles are now understood to play a crucial role in regulating apoptosis, and their dysfunction can lead to cell death. Mitochondria are also crucial for the tricarboxylic acid (TCA) cycle and fatty acid beta-oxidation (FAO). Therefore, we examined the mRNA expression of key molecules involved in these pathways (Citrate synthase (CS) and isocitrate dehydrogenase 2 (IDH2) for the tricarboxylic acid cycle, and Acyl CoA synthase long chain family member 1 (ACSL1) and Carnitine palmitoyltransferase 2 (CPT2) for fatty acid beta-oxidation) in SNU-449 and HepG2 cells treated with various treatments (control, DSF, Cu^2+^, and DSF/Cu). As shown in **Figure 1E-L**, the mRNA expression of CS, IDH2, ACSL1, and CPT2 was downregulated in the DSF/Cu treatment group, indicating impaired mitochondrial function.

**Figure 2:**
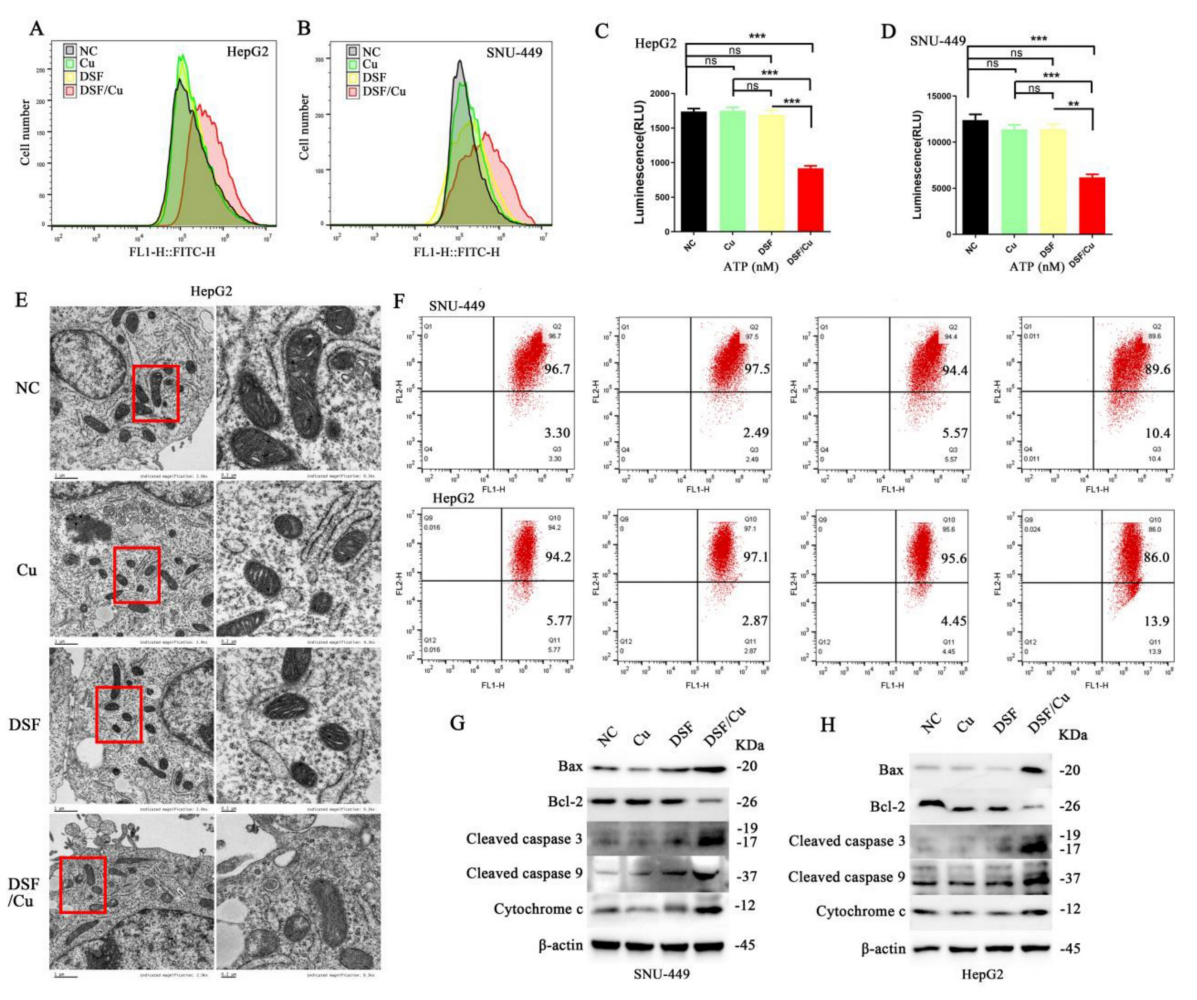
** Disulfiram and copper disrupted mitochondrial function and induced apoptosis in HCC cells.** A and B. Administration of DSF/Cu in HCC cell lines (HepG2 and SNU-449) increased ROS generation. C and D. DSF/Cu treatment reduced ATP production in both cell lines. E. DSF/Cu disrupted mitochondrial morphology in HepG2 cells. F. DSF/Cu induced a decrease in mitochondrial membrane potential. G and H. DSF/Cu downregulated Bcl-2 expression, upregulated Bax expression, and increased levels of cleaved caspase 3, cleaved caspase 9, and Cytochrome c in HCC cell lines (HepG2 and SNU-449). *p < 0.05, **p < 0.01, ***p < 0.001. ATP: adenosine triphosphate, HCC: Hepatocellular carcinoma, ROS: reactive oxygen species.

To further assess mitochondrial function, ROS generation was quantified in HepG2 and SNU-449 cell lines using the DCFH-DA probe. A significant increase in ROS production was observed within the DSF/Cu group compared to the control group (**Figure 2A and 2B**). Subsequent assessment of ATP levels revealed a significant decrease in ATP levels in the DSF/Cu group relative to controls (**Figure 2C and 2D**). Moreover, JC-1 staining revealed a significant decrease in MMP in cells following DSF/Cu treatment compared to controls (**Figure 2F**). Finally, transmission electron microscopy (TEM) revealed alterations in mitochondrial ultrastructure, characterized by the loss of mitochondrial cristae in the DSF/Cu group (**Figure 2E**). These findings collectively demonstrate that the DSF/Cu combination induces mitochondrial dysfunction in hepatocellular carcinoma cell lines (HepG2 and SNU-449).

To explore the underlying mechanisms of apoptosis, we investigated the expression levels of key proteins involved in the mitochondrial apoptotic pathway. Our analysis revealed an upregulation of Bax, cytochrome c, cleaved caspase-3 and -9, along with a downregulation of B-cell lymphoma-2 (Bcl-2) within the DSF/Cu combination treatment group (**Figure 2G and 2H**). In summary, our findings substantiate that the DSF/Cu combination triggers mitochondrial dysfunction in HCC cells, ultimately leading to apoptosis-mediated cell death.

### DSF/Cu triggered mitochondrial damage and apoptosis in HCC cells by upregulating ATF3

**Figure 3:**
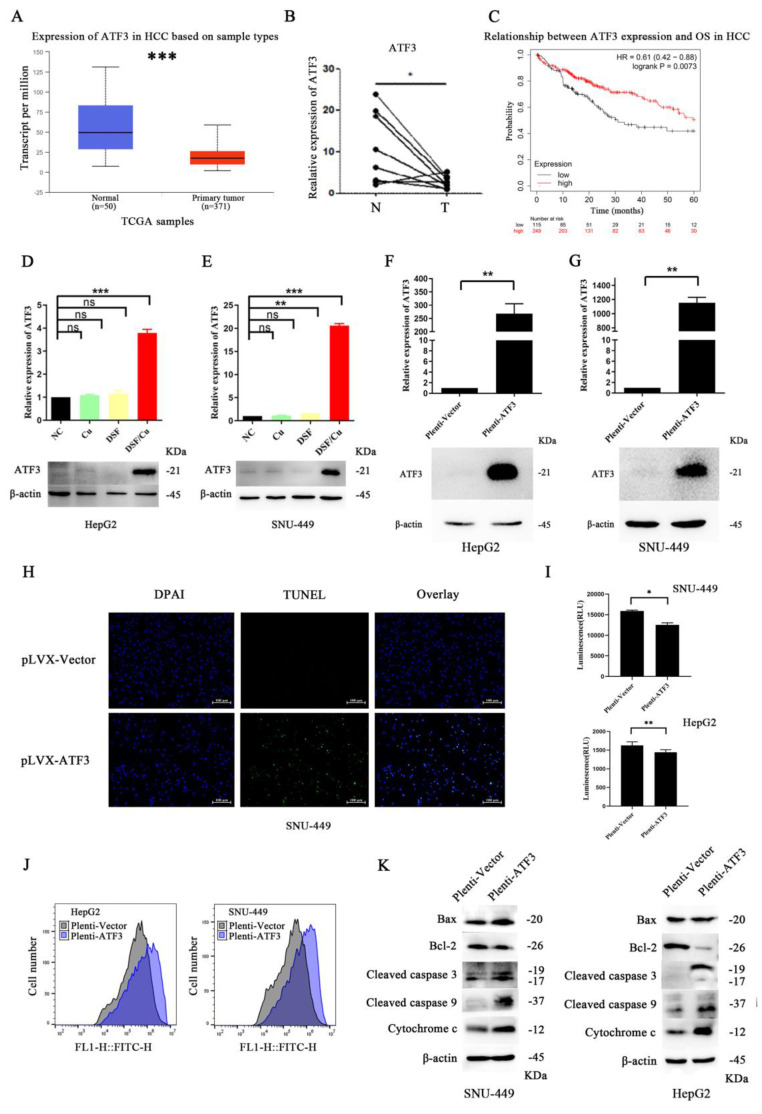
** ATF3 expression in HCC and its impact on cellular processes.** A. Analysis of TCGA data showed downregulation of ATF3 expression in HCC. B. Validation of decreased ATF3 expression in paired HCC tissues. C. High ATF3 expression correlated with a better prognosis. D and E. Treatment with DSF/Cu in liver cancer cell lines (HepG2 and SNU-449) resulted in significant upregulation of ATF3 expression. F and G. Verification of the effects of ATF3 overexpression plasmids in liver cancer cell lines (HepG2 and SNU-449). H. ATF3 overexpression in the SNU-449 liver cancer cell line led to increased cell apoptosis. I. ATF3 overexpression reduced ATP production in liver cancer cell lines (HepG2 and SNU-449). J. ATF3 overexpression increased ROS generation in liver cancer cell lines (HepG2 and SNU-449). K. ATF3 overexpression downregulated Bcl-2 expression and upregulated Bax expression, cleaved caspase 3, cleaved caspase 9, and cytochrome c expression in liver cancer cell lines (HepG2 and SNU-449). *p < 0.05, **p < 0.01, ***p < 0.001. ATF3: activating transcription factor 3, ATP: adenosine triphosphate, HCC: Hepatocellular carcinoma, ROS: reactive oxygen species.

To gain a deeper understanding of the specific mechanism by which DSF/Cu induces mitochondrial damage and apoptosis in hepatocellular carcinoma cells, we employed a comprehensive multi-step approach (specific methods are described in the supplementary materials). Our results revealed that ATF3 may play a crucial role in DSF-mediated treatment of HCC cell lines (differential expression analysis and protein-protein interaction (PPI) network analysis results are presented in **Supplementary Figure 1**). Analysis of The Cancer Genome Atlas (TCGA) liver cancer data using the UALCAN platform revealed that ATF3 expression was significantly downregulated in HCC compared to normal tissues (**Figure 3A**) (**detailed methods are provided in the supplementary materials**), a finding further validated in HCC tissue samples (**Figure 3B**). Moreover, lower ATF3 expression correlated with a worse prognosis, with a significant difference in overall survival (*p*=0.0073) (**Figure 3C**). Furthermore, HCC samples from TCGA were stratified based on median ATF3 expression levels and a functional enrichment analysis of differentially expressed genes between the high- and low-expression groups was conducted (**detailed methods are provided in the supplementary materials**). The results revealed that ATF3 was associated with energy metabolism, mitochondrial function, and cell apoptosis (**Supplementary Figure 2**).

Based on the above results, we hypothesized that DSF/Cu induces mitochondrial damage and apoptosis by upregulating ATF3. To validate this hypothesis, we compared ATF3 expression in HepG2 and SNU-449 cell lines treated with DSF/Cu to the other treatment groups. Our results showed that DSF/Cu treatment significantly increased ATF3 expression (**Figure 3D and 3E**). To further validate this hypothesis, we generated a plasmid overexpressing ATF3 and confirmed its transfection efficiency (**Figure 3F and 3G**). TUNEL assay results (**Figure 3H**) indicated increased apoptosis in the SNU-449 cell line with ATF3 overexpression. Furthermore, overexpression of ATF3 in both cell lines resulted in downregulation of CS, IDH2, ACSL1, and CPT2 expression (**Supplementary Figure 3A**), decreased ATP production (**Figure 3I**), and increased ROS generation (**Figure 3J**). Oil Red O staining experiments revealed that ATF3 overexpression led to lipid accumulation in HCC cell lines (**Supplementary Figure 3B**). Besides, ATF3 overexpression in HepG2 cells decreased the MMP (**Supplementary Figure 3C**). Furthermore, ATF3 overexpression led to the upregulation of mitochondrial apoptosis-related mediators (Bax, Cytochrome c, cleaved caspase-3 and -9) and downregulation of Bcl-2 (**Figure 3K**).

### Validation of ATF3-mediated mitochondrial damage and apoptosis in HCC cells

**Figure 4:**
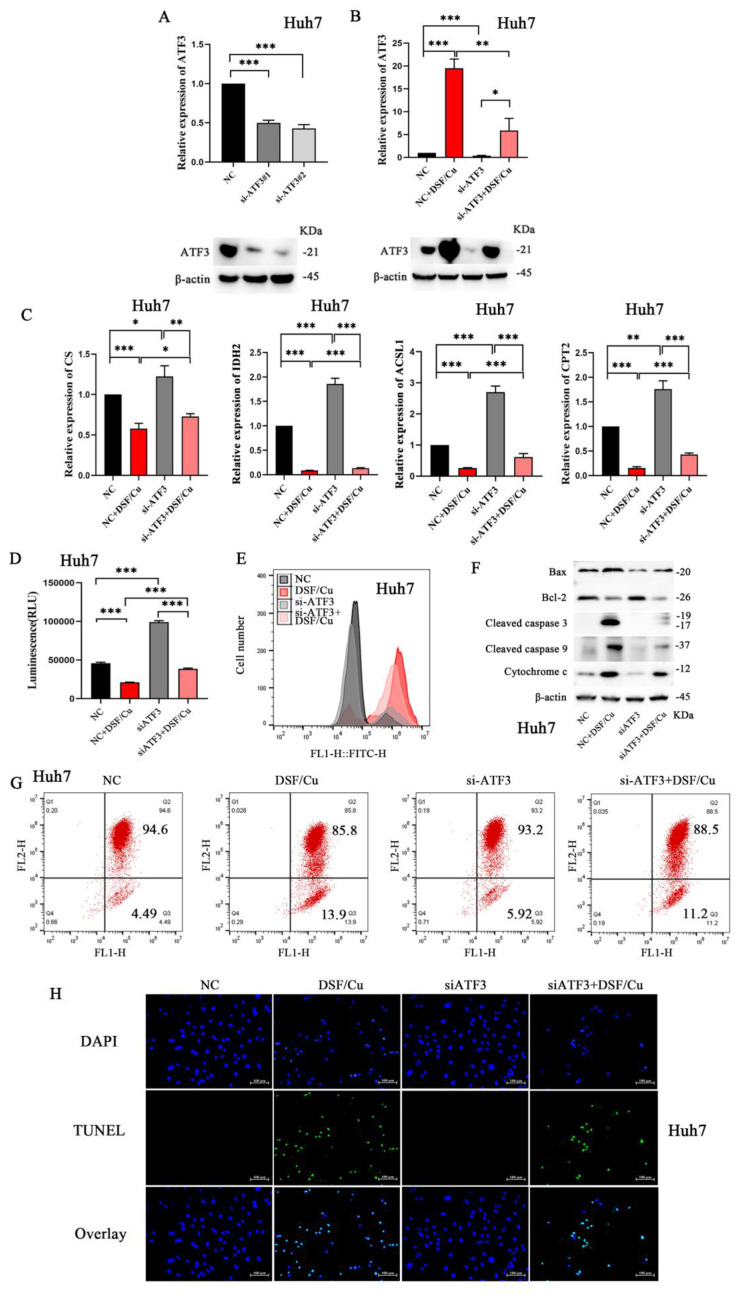
** ATF3-mediated mitochondrial damage and cell apoptosis in HCC cells.** A. Construction of si-ATF3 and validation of its silencing effect in the Huh7 cell line. B. Validation of ATF3 knockdown and its response to DSF/Cu treatment. ATF3 expression levels were assessed in Huh-7 cells across four treatment groups: NC, DSF/Cu alone, siRNA-mediated ATF3 knockdown (a pool of siATF3#1 and #2), and the combination of knockdown with DSF/Cu. C. si-ATF3 transfection increased the protein expression of CS, IDH2, ACSL1, and CPT2 in Huh7 cells, whereas DSF/Cu treatment in si-ATF3-transfected cells reversed this effect, leading to downregulation of these proteins. D. si-ATF3 transfection led to increased ATP production in Huh7 cells compared to the control group. Conversely, DSF/Cu treatment in si-ATF3-transfected cells significantly decreased ATP production. E. While DSF/Cu treatment alone increased ROS generation, the combination of si-ATF3 and DSF/Cu resulted in ROS suppression in Huh7 cells. F. Compared to the NC group, si-ATF3 transfection alone upregulated the anti-apoptotic protein Bcl-2 and downregulated the pro-apoptotic proteins Bax and Cytochrome c. In contrast, the addition of DSF/Cu to si-ATF3-transfected cells reversed this pattern by downregulating Bcl-2 and further upregulating Bax, Cytochrome c, Cleaved caspase-9, and Cleaved caspase-3. G. DSF/Cu treatment in si-ATF3-transfected Huh7 cells exhibited a decrease in mitochondrial membrane potential compared to control and si-ATF3 alone. H. DSF/Cu treatment induced apoptosis in Huh7 cells; however, this effect was significantly attenuated when ATF3 was silenced (via si-ATF3). *p < 0.05, **p < 0.01, ***p < 0.001. ATF3: activating transcription factor 3, ATP: adenosine triphosphate, ACSL1: Acyl-CoA Synthetase Long Chain Family Member 1, HCC: Hepatocellular carcinoma, ROS: reactive oxygen species, CS: Citrate Synthase, IDH2: Isocitrate Dehydrogenase 2, and CPT2: Carnitine Palmitoyl Transferase 2.

To further validate our findings, Huh-7 cells were selected for subsequent experiments due to their relatively higher ATF3 expression, as observed in **Supplementary Figure 3D**. We designed and validated the knockdown efficiency of two small interfering RNAs (siRNAs) targeting ATF3 (**Figure 4A**, sequences provided in **Supplementary Table 3**). Following siATF3 transfection, DSF/Cu treatment (siATF3+DSF/Cu group) induced significant upregulation of ATF3 expression (**Figure 4B**). Huh-7 cells were then subjected to various treatments: DSF/Cu alone, siATF3 alone, and their combination (siATF3+DSF/Cu). As expected, DSF/Cu treatment resulted in the downregulation of CS, IDH2, ACSL1, and CPT2, while siATF3 treatment yielded contrasting results (**Figure 4C**). The combination of siATF3 and DSF/Cu consistently induced downregulation of CS, IDH2, ACSL1, and CPT2. Furthermore, compared to the control group, siATF3 transfection in Huh-7 cells resulted in increased ATP production (**Figure 4D**). Conversely, treatment with DSF/Cu following siATF3 transfection led to decreased ATP production. Comparative analysis revealed that while DSF/Cu treatment alone increased ROS generation relative to siATF3 treatment alone, the combined siATF3+DSF/Cu intervention paradoxically suppressed ROS generation (**Figure 4E**). Moreover, DSF/Cu treatment significantly upregulated pro-apoptotic markers, including Bax, Cytochrome c, Cleaved caspase 3, and Cleaved caspase 9 (**Figure 4F**). Conversely, compared to the DSF/Cu treatment alone, the combination treatment (siATF3+DSF/Cu) downregulated the expression of these pro-apoptotic markers. Consistent with these findings, DSF/Cu treatment resulted in a significant decrease in MMP compared to siATF3 treatment alone. However, no significant difference in MMP reduction was observed between the DSF/Cu treatment group and the combination treatment group (siATF3 + DSF/Cu) (**Figure 4G**). Administration of DSF/Cu induced apoptosis in Huh-7 cells; however, this effect was slightly attenuated by co-treatment with si-ATF3 (**Figure 4H**). These results collectively suggest that ATF3 knockdown can partially prevent the deleterious effects of DSF/Cu on mitochondrial function and apoptosis.

### *In vivo* validation of the tumor-inhibitory effects of DSF/Cu

**Figure 5:**
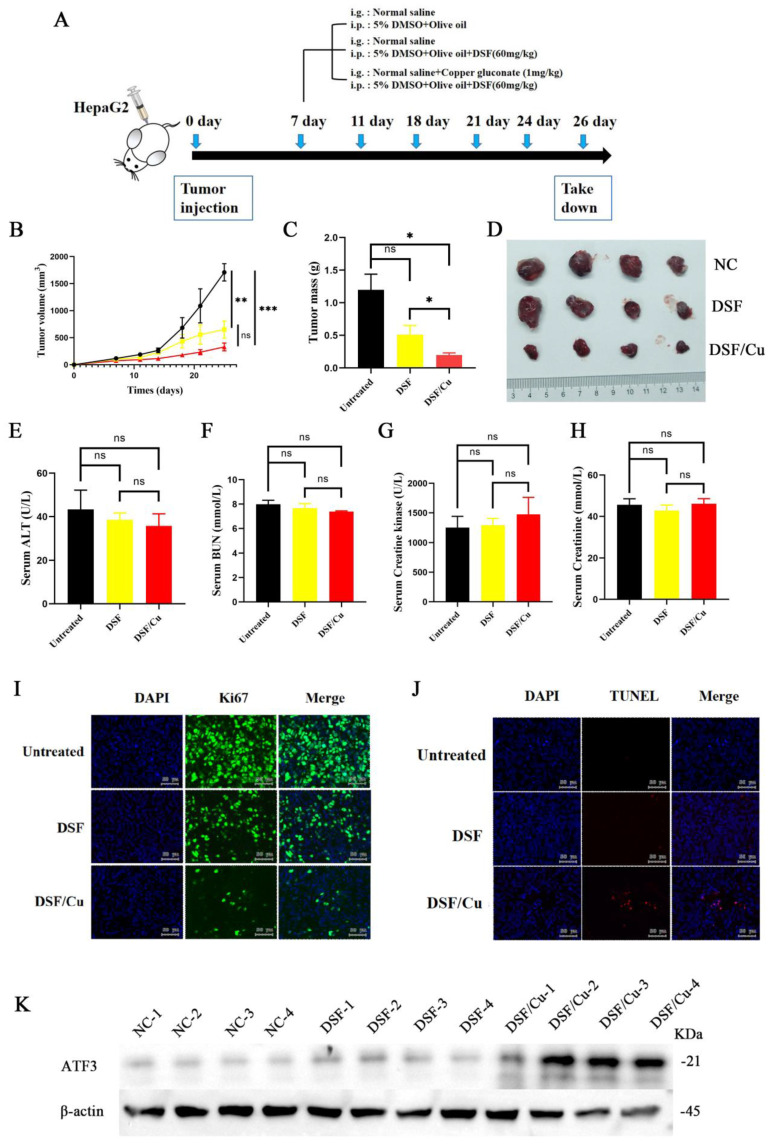
** DSF/Cu treatment inhibited tumor growth, promoted apoptosis, and upregulated ATF3 *in vivo*.** A. Schematic representation of the experimental design for the *in vivo* studies evaluating the effects of DSF/Cu on tumor growth and apoptosis. B. Tumor growth curve depicting changes in tumor size over time across different treatment groups. C.Tumor volume quantification at the endpoint of the experiment. The combination treatment (DSF/Cu) significantly reduced tumor volume compared to the control group. D. Representative images of tumors isolated from mice in each treatment group at the experimental endpoint. E-H. Analysis of liver, kidney and cardiac/muscle function in mice: plasma levels of ALT, BUN, Cr, and CK following treatment with control or DSF/Cu. I. Immunohistochemical analysis of Ki-67 expression in tumor tissues from each group. Ki-67 is a marker of cell proliferation. J. TUNEL staining of tumor tissues from each group to assess apoptosis. K. Western blot analysis of ATF3 protein expression levels in tumor tissues from each group. *p < 0.05, **p < 0.01, ***p < 0.001. ALT: alanine aminotransferase, ATF3: activating transcription factor 3, BUN: blood urea nitrogen, Cr: creatinine, and CK: creatinine kinase.

HepG2 cells were implanted into five-week-old male NOD/ShiLtJGpt-*Prkdc^em26Cd52^Il2rg ^em26Cd22^* /Gpt mice, which were randomized into three different treatment groups: vehicle control, DSF alone, and DSF/Cu (experimental flowchart in **Figure 5A**). The DSF/Cu treatment group exhibited slower tumor growth (**Figure 5B**) and markedly reduced tumor volumes (**Figure 5C and 5D**) compared with the control group. DSF/Cu treatment showed no adverse effects on liver, kidney, and heart function in mice (**Figure 5E-H**). Further analysis revealed that DSF/Cu treatment inhibited tumor cell proliferation (**Figure 5I**) and increased apoptosis (**Figure 5J**). Finally, immunoblot analysis of tumor tissues demonstrated significant upregulation of ATF3 in the DSF/Cu group (**Figure 5K**). These findings collectively suggest that DSF/Cu may promote mitochondrial damage and induce apoptosis in hepatocellular carcinoma cells by upregulating ATF3.

### DSF/Cu combined with lenvatinib demonstrated superior anti-tumor effects

**Figure 6:**
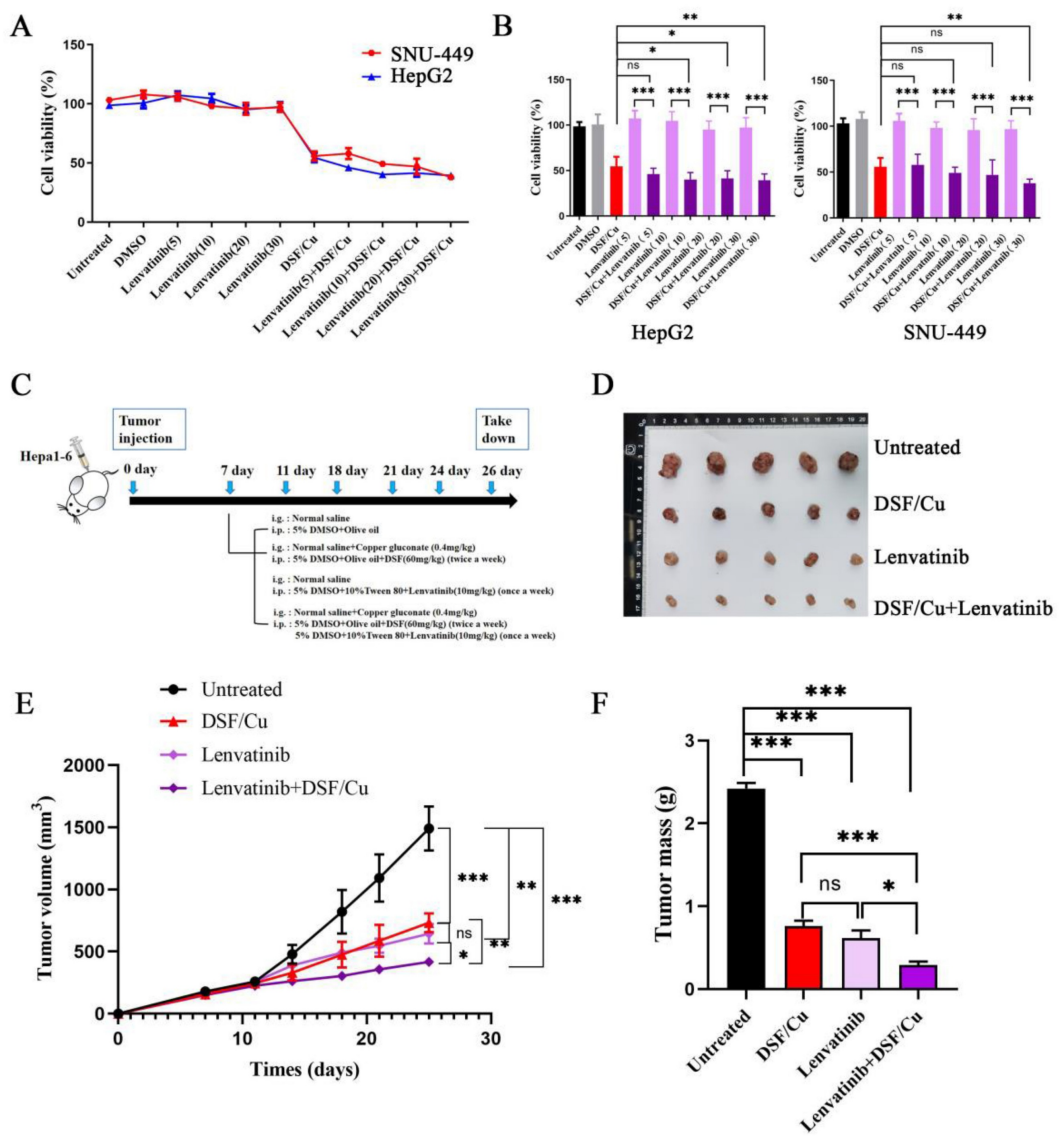
**DSF/Cu enhanced the anti-tumor effect of lenvatinib *in vitro* and *in vivo*.** A. Liver cancer cell lines (HepG2 and SNU-449) were treated with lenvatinib (Lenv.), DSF/Cu, or the combination (Lenv. + DSF/Cu) for 24 hours, and cell viability was assessed. The combination treatment resulted in significantly greater inhibition of cell viability compared to lenvatinib alone. B. Bar graphs corresponding to the data from panel A, showing the viability of HepG2 and SNU-449 cells under the indicated treatments. C. Schematic representation of the *in vivo* experiment design to evaluate the combined effects of DSF/Cu and lenvatinib on tumor growth in mice. D. Representative images of tumors isolated from mice in each treatment group during the experiment. E. Tumor growth curve depicting changes in tumor size over time across different treatment groups. F. Tumor volume quantification at the endpoint of the experiment. The combination treatment (Lenv. + DSF/Cu) significantly reduced tumor volume compared to other groups (*p < 0.05, **p < 0.01, ***p < 0.001).

Given the established efficacy of lenvatinib in treating HCC, its high cost presents a significant barrier to patient access. Therefore, we investigated whether combining DSF/Cu with lenvatinib could enhance HCC suppression, aiming to achieve a more cost-effective therapeutic approach. In initial experiments, HCC cell lines were treated with the following: (i) DSF/Cu alone, (ii) varying concentrations of lenvatinib, and (iii) a lenvatinib-DSF/Cu combination. Comparative analysis revealed that the lenvatinib-DSF/Cu combination exhibited significantly greater suppression of HCC cell viability than lenvatinib monotherapy (**Figure 6A-B**).

To further evaluate the anti-tumor efficacy of the combination treatment (lenvatinib+DSF/Cu), a subcutaneous xenograft model was established using Hepa1-6 cells implanted in five-week-old male NOD/ShiLtJGpt-*Prkdc^em26Cd52^Il2rg ^em26Cd22^* /Gpt mice (**Figure 6C**). Our findings validated the anti-tumor efficacy of DSF/Cu monotherapy compared to vehicle controls (**Supplementary Figure 4**). For the combination therapy evaluation, mice were divided into four groups: control, DSF/Cu alone, lenvatinib alone, and lenvatinib-DSF/Cu combination. The combination treatment group exhibited significantly slower tumor growth (**Figure 6E**) and reduced tumor volumes (**Figure 6D and 6F**) compared to the other groups. These findings collectively highlight the superior anti-tumor effect of the lenvatinib-DSF/Cu combination compared to either treatment alone, suggesting a potential synergistic effect against HCC.

## Discussion

Hepatocellular carcinoma is a significant global health burden, characterized by high mortality rates. Data from GLOBOCAN 2020 indicate that HCC ranks as the sixth most prevalent cancer worldwide and the third leading cause of cancer-related deaths, contributing to approximately 830,000 fatalities annually 1. The limited treatment options for HCC are attributed to late-stage diagnosis, ineffective therapies, and high rates of recurrence post-intervention 2. Conventional therapeutic approaches such as surgery, chemotherapy, and radiotherapy are often hindered by drug resistance, associated toxicities, and limited effectiveness against advanced stages of HCC 10. Repurposing existing FDA-approved drugs for the treatment of HCC offers a promising avenue. Indeed, these drugs possess established safety profiles, well-characterized pharmacokinetics, and the potential for cost savings compared with the development of entirely new drugs 11. DSF, initially approved for the treatment of alcohol dependence, has demonstrated encouraging anti-cancer potential in both preclinical and clinical studies, particularly when combined with copper ions (Cu^2+^) 12. Recent studies have revealed that DSF/Cu affects the progression and treatment of various tumors through different mechanisms 13. For instance, growing evidence suggests that DSF/Cu can induce ferroptosis and exert anti-tumor effects by modulating the tumor immune microenvironment through the activation of the cGAS-STING innate immunity pathway and the induction of immunogenic cell death 14-16.

Notably, elevated copper levels have been observed in the serum and tissues of various human cancers 17. This perspective proposes exploring DSF as a potential therapeutic target for tumors by specifically binding to copper within tumor tissues. Repurposing FDA-approved drugs such as DSF/Cu for HCC treatment represents a critical strategy in addressing the complexities of this disease 18. Such approaches leverage existing therapeutic agents while simultaneously paving the way for the development of innovative and more effective treatments, ultimately aiming to improve patient outcomes.

Our study substantiated that the significant anti-tumor effects of DSF/Cu against HCC were primarily mediated by mitochondrial dysfunction. DSF/Cu treatment disrupted key metabolic pathways in HCC cells, evidenced by dysregulation of the TCA cycle and fatty acid β-oxidation components, accompanied by elevated ROS production, diminished ATP generation, loss of mitochondrial membrane potential, and ultrastructural abnormalities. As the liver is a highly metabolic organ densely populated with mitochondria 19, these findings collectively establish mitochondrial impairment as a central mechanism of DSF/Cu's anti-HCC activity. While prior studies have emphasized that DSF/Cu induces ferroptosis and cuproptosis in the treatment or progression of cancers 20, 21, our work reveals a novel ATF3-mediated pathway through which DSF/Cu induces mitochondrial damage and subsequent apoptosis, ultimately suppressing HCC progression. The resulting severe mitochondrial dysfunction can lead to impaired energy production, heightened oxidative stress, and alterations in metabolic pathways, all contributing to tumor suppression 22, 23. Besides, damaged mitochondria can trigger apoptotic pathways, leading to the elimination of cancerous cells and inhibition of tumor growth 24. Similarly, our study found that treatment with DSF/Cu resulted in the downregulation of Bcl-2 and upregulation of Bax, Cytochrome c, Cleaved caspase 3, and 9 in hepatocellular carcinoma cell lines, indicating that DSF/Cu not only induced mitochondrial damage dysfunction but also promoted HCC cell apoptosis, thereby inhibiting tumor progression.

ATF3, a transcription factor known for its role in stress responses and inflammation 25, 26, has recently emerged as a potential target in cancer therapy. Importantly, the present study provides compelling evidence for a novel mechanism by which DSF/Cu treatment upregulates ATF3, leading to mitochondrial dysfunction and apoptosis in HCC cells. Research suggests that ATF3 promotes apoptosis by binding to the promoters of RIPK3 and Caspase-9, leading to their upregulation. Moreover, it can enhance the transcription of Caspase-3 and Bad by activating FOXO3a, thereby regulating cell death pathways 27. Interestingly, our study found that DSF/Cu treatment upregulated ATF3 expression in HCC cell lines, associated with increased ROS generation, mitochondrial damage, and cell apoptosis. This aligned with observations by Chen *et al.*, who reported that ATF3 significantly inhibited HCC cell proliferation and mobility 8. Our research revealed that DSF/Cu induced mitochondrial damage and promoted apoptosis in HCC cells via ATF3 upregulation. This finding not only enhances our understanding of ATF3's role in mediating mitochondrial damage and apoptosis but also provides valuable insights into its contribution to HCC pathogenesis and progression.

The present study systematically evaluated the comparative efficacy of lenvatinib monotherapy (a clinically approved antiangiogenic treatment for HCC) versus combination therapy with DSF/Cu in both *in vitro* and *in vivo* models 28. The marked anti-tumor efficacy of the DSF/Cu and lenvatinib combination, compared to lenvatinib alone, supports its further investigation as a promising clinical candidate for improving patient outcomes. Such an approach might allow for reduced lenvatinib dosage, thereby lowering the economic burden on patients, while simultaneously achieving improved therapeutic efficacy through synergistic antitumor activity.

Despite its findings, this study is subject to the limitations typical of preclinical research. *In vitro* cell experiments, while valuable, cannot fully recapitulate the intricate complexities of the tumor microenvironment *in vivo*. Moreover, potential discrepancies exist between the pathophysiology of mouse models and human HCC. Furthermore, the partial rescue of the apoptotic phenotype, as assessed by TUNEL assay, in the si-ATF3 + DSF/Cu group suggests that DSF/Cu-induced apoptosis is multifactorial and not solely dependent on ATF3 signaling. These findings collectively highlight the complexity of DSF/Cu-induced apoptosis and underscore the need for further investigation using broader molecular targets and more advanced *in vivo* models.

Indeed, these limitations will be addressed in future research to facilitate the translation of findings into clinically relevant therapeutic strategies. A nanomaterial-based delivery system encapsulating both DSF and copper ions will be developed, with the dual objectives of enhancing anti-tumor efficacy and mitigating potential hepatic metabolic complications 29. Besides, the mechanisms underlying the synergistic anti-tumor effects of DSF and copper ions warrant further research. Experimental validation will be extended to include a more comprehensive panel of HCC cell lines and advanced animal models. Furthermore, rigorous safety evaluations and dose optimization studies are essential to ensure the safe and effective translation of DSF/Cu combination therapy into clinical practice.

**Figure 7:**
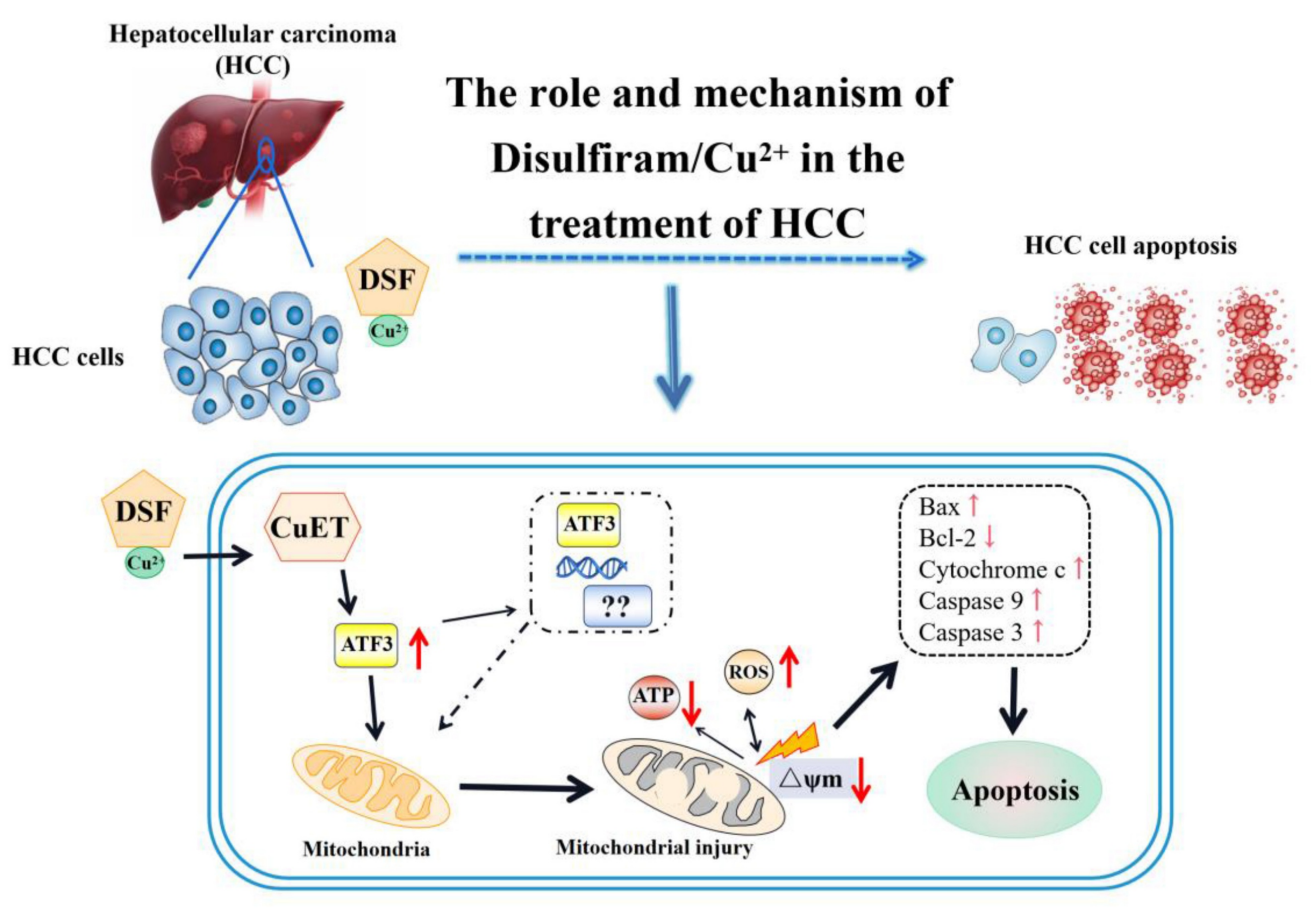
** Mechanism of ATF3-mediated mitochondrial dysfunction and apoptosis induction by DSF/Cu in hepatocellular carcinoma.** ATF3: activating transcription factor 3, ATP: adenosine triphosphate, CuET: bis (diethyldithiocarbamate)-copper, HCC: Hepatocellular carcinoma, ROS: reactive oxygen species.

In conclusion, this study presents hitherto undocumented evidence that DSF/Cu treatment upregulates ATF3 expression in hepatocellular carcinoma cells. This upregulation contributes to mitochondrial dysfunction and the induction of apoptosis, ultimately leading to the inhibition of HCC cell proliferation (**Figure 7**). Furthermore, the combination of DSF/Cu with lenvatinib exhibits superior inhibitory effects on HCC compared to lenvatinib alone. Taken together, these findings hold promise for the development of novel therapeutic strategies for this patient population.

## Supplementary Material

Supplementary methods, figures and tables.

## Abbreviations

ACSL1: Acyl CoA synthase long chain family member 1
ALT: alanine aminotransferase
ATP: adenosine triphosphate
ATF3: activating transcription factor 3
BUN: blood urea nitrogen
CK: creatinine kinase
CPT2: Carnitine palmitoyltransferase 2
Cr: creatinine
CREB: cAMP-responsive element-binding protein
CS: Citrate synthase
Cu: copper
Cu^2+^: copper ions
DSF: disulfiram
DSF/Cu: disulfiram/copper complex
DCFH-DA: 2',7'-dichlorofluorescein diacetate
FAO: fatty acid beta-oxidation
FDA: Food and Drug Administration
GEO: Gene Expression Omnibus
HCC: Hepatocellular carcinoma
ICIs: immune checkpoint inhibitors
IDH2: isocitrate dehydrogenase 2
Lenv.: lenvatinib
MMP: mitochondrial membrane potential
PPI: protein-protein interaction
ROS: reactive oxygen species
siRNAs: small interfering RNAs
STR: short tandem repeat
TCA cycle: tricarboxylic acid cycle
TCGA: The Cancer Genome Atlas
TEM: transmission electron microscopy
