# Supplementary material for: Disulfiram/Copper Combination as a Potential Therapeutic Approach for Hepatocellular Carcinoma: Targeting the ATF3-Mitochondrial Cell Death Pathway
Source: J Cancer. 2026 Jan 1;17(1):117–30. doi: 10.7150/jca.113442 (PMC12719560; doi:10.7150/jca.113442)
Supplement: Supplementary file 1 — Supplementary methods, figures and tables. [file jcav17p0117s1.pdf]

Experimental methods

Real-time qPCR

Following treatment with specific agents for 24 hours, cells were subjected to total RNA extraction using TRIzol reagent (15596018, Ambion, Thermo Fisher Scientific, USA). The purity and concentrations of the RNA were assessed using a Nanodrop 2000 spectrophotometer (Thermo Fisher Scientific, USA). Subsequently, 1 μg of total RNA was reverse transcribed into cDNA using a reverse transcription kit (FSQ-101, TOYOBO, Japan). Target gene amplification was conducted on a LightCycler® 480 System with LightCycler® 480 SYBR Green I Master, following the manufacturer's instructions. The relative transcript levels of the target genes were calculated using the $2^{-\Delta\Delta CT}$ method, with the *actin* gene serving as the reference. The primer sequences used are detailed in Supplementary Table 1.

Western blotting analysis

Tumor tissues and cells were lysed with RIPA lysate on ice for 30 minutes. The lysates were then sonicated and centrifuged at 12,000×g for 15 minutes at 4°C to obtain total protein in the supernatant. Protein quantification was performed using the BCA protein assay kit (P0012, Beyotime, China). The protein extracts were boiled in 5×sodium dodecyl sulfate (SDS) sample loading buffer for 10 minutes to facilitate protein denaturation. The samples underwent sodium dodecyl sulfate-polyacrylamide gel electrophoresis (SDS-PAGE) separation and were subsequently transferred to a polyvinylidene fluoride membrane (ISEQ00010, PVDF, Merck Millipore, Germany). These membranes were blocked with 5% non-fat milk or BSA for 1 hour and then incubated overnight with specific primary antibodies. After washing the membranes three times with PBST buffer, they were incubated with the corresponding secondary antibodies for 1 hour at room temperature. Protein bands were visualized using the Chemiluminescence ECL kit (MA0186, Meilunbio, Dalian, China). The intensity of protein bands was analyzed using β-actin as a control and Image J software (NIH Image, Bethesda, MD, USA). The antibodies used are detailed in Supplementary Table 2.

Oil Red O Staining

Cells were seeded in 12-well plates at a density of 1×10^5 cells per well and subjected to various treatments. Lipid accumulation was then assessed using an Oil Red O staining kit (G1262, Solarbio, China). The cells were washed twice with phosphate-buffered saline (PBS) and fixed

with 4% paraformaldehyde for 30 minutes at room temperature. After fixation, the cells were washed again with PBS and stained with Oil Red O solution following the manufacturer's instructions. The staining solution was added to each well and incubated for 15-30 minutes at room temperature, followed by rinsing with distilled water to remove excess dye. The cells were then stained with Mayer's hematoxylin solution and incubated for 15-30 seconds at room temperature, followed by rinsing with distilled water to remove excess dye. Finally, the stained cells were observed under a light microscope or captured using an imaging system.

Bioinformatics analysis and ATF3 functional enrichment analysis

Gene expression data were obtained from the Gene Expression Omnibus (GEO) database, specifically dataset GSE42318, to conduct differential expression analysis in HCC cell lines (Huh7 and Huh1) treated with DSF. The dataset was selected due to its relevance to our study' s focus on the effects of DSF treatment in HCC cells and the availability of gene expression data related to oxidative stress and apoptosis pathways. Differential gene expression analysis was conducted using R, and protein-protein interaction (PPI) networks were constructed using STRING (https://string-db.org/). Hub genes were analyzed by constructing a PPI network in Cytoscape software (version 3.7.2). The network was subsequently processed using the MCODE plugin for module detection and the CytoHubba plugin for hub gene identification. MCODE was used to screen the modules of the PPI network identified by degree cutoff =2, node score cutoff =0.2, K core =2 and a maximum depth=100. The importance of these hub genes was assessed using the Cytoscape plugin CytoHubba, employing the topological algorithm "degree". Besides, the MCODE plugin and the Maximal Clique Centrality (MCC) algorithm identified ATF3 as a key molecule. TCGA is the largest and most widely used public resource providing genomic data, including somatic mutations, gene expression, gene methylation profiles, and copy number variation (CNV) datasets for thousands of tumor samples [1]. Subsequently, using liver carcinoma (LIHC) samples from TCGA (n=365 cases), we stratified patients into high- and low-expression groups based on the median ATF3 expression level. Differential gene expression analysis was performed between these groups, followed by functional enrichment analyses using GO, KEGG, and Reactome databases. TCGA was used to analyze ATF3 expression in normal and LIHC samples. In this study, UALCAN (http://ualcan.path.uab.edu)[2], an interactive web portal for analyzing cancer transcriptome

data, was utilized to investigate ATF3 expression in liver cancer using TCGA datasets. The analysis involved comparing ATF3 expression levels between tumor and normal liver tissues. The Kaplan-Meier Plotter (http://www.kmplot.com) is an online database that can assess the effects of 54,675 genes on survival using 10,461 cancer samples[3]. In this study, this tool was used to analyze overall survival in patients with HCC.

References:

1. Deng M, Brägelmann J, Schultze JL, Perner S: Web-TCGA: an online platform for integrated analysis of molecular cancer data sets. BMC Bioinformatics 2016, 17:72.
2. Chandrashekar DS, Bashel B, Balasubramanya SAH, Creighton CJ, Ponce-Rodriguez I, Chakravarthi B, Varambally S: UALCAN: A Portal for Facilitating Tumor Subgroup Gene Expression and Survival Analyses. Neoplasia 2017, 19(8):649-658.
3. Mongre RK, Jung S, Mishra CB, Lee BS, Kumari S, Lee MS: Prognostic and Clinicopathological Significance of SERTAD1 in Various Types of Cancer Risk: A Systematic Review and Retrospective Analysis. Cancers (Basel) 2019, 11(3).

Supplementary Figures

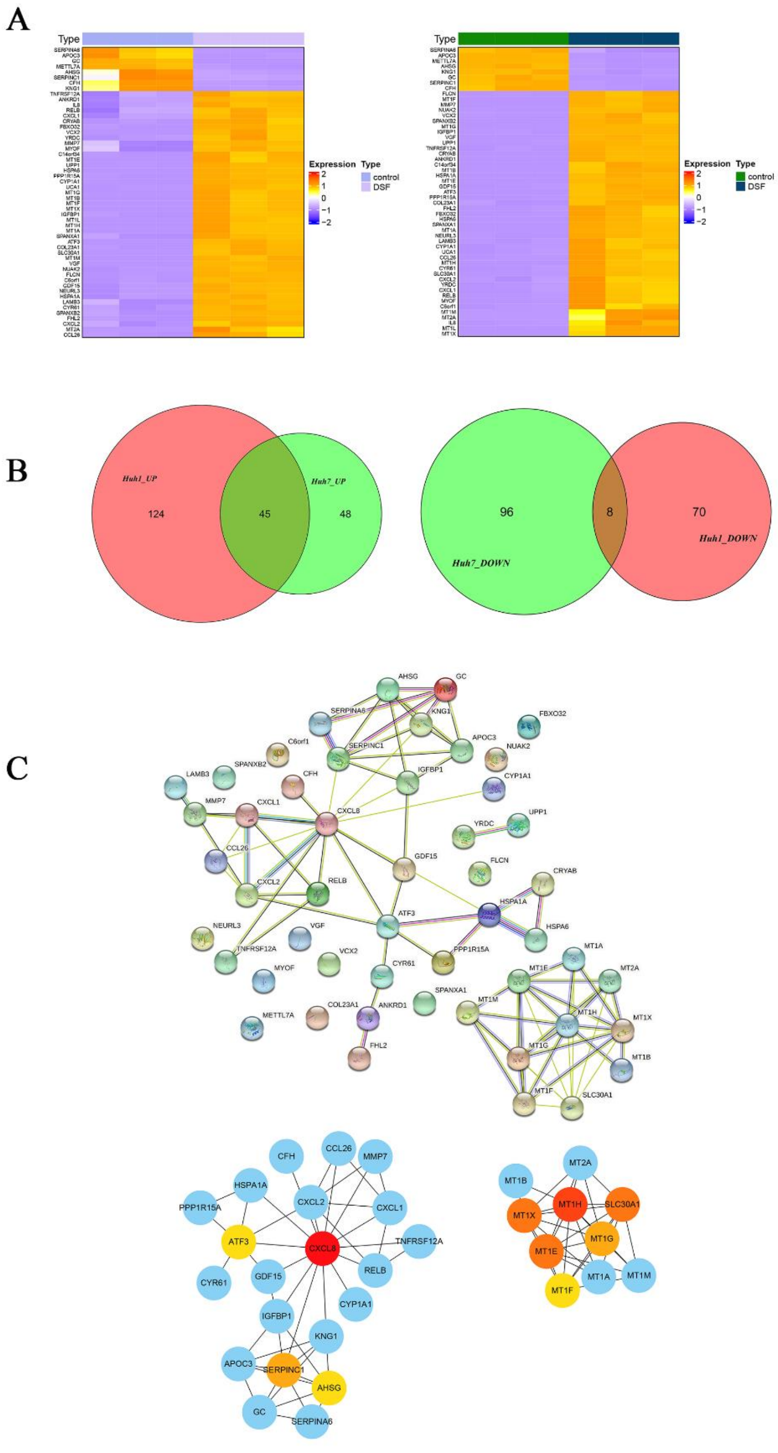

Supplementary Figure 1. Identification of differentially expressed genes (DEGs) in HCC cell lines following DSF treatment using the GEO database. A. Heatmaps of differentially expressed genes in different cell lines. B. Venn diagram showing the overlap of upregulated and downregulated genes between two cell lines. C. ATF3 was identified as a key protein through protein-protein interaction (PPI) network analysis.

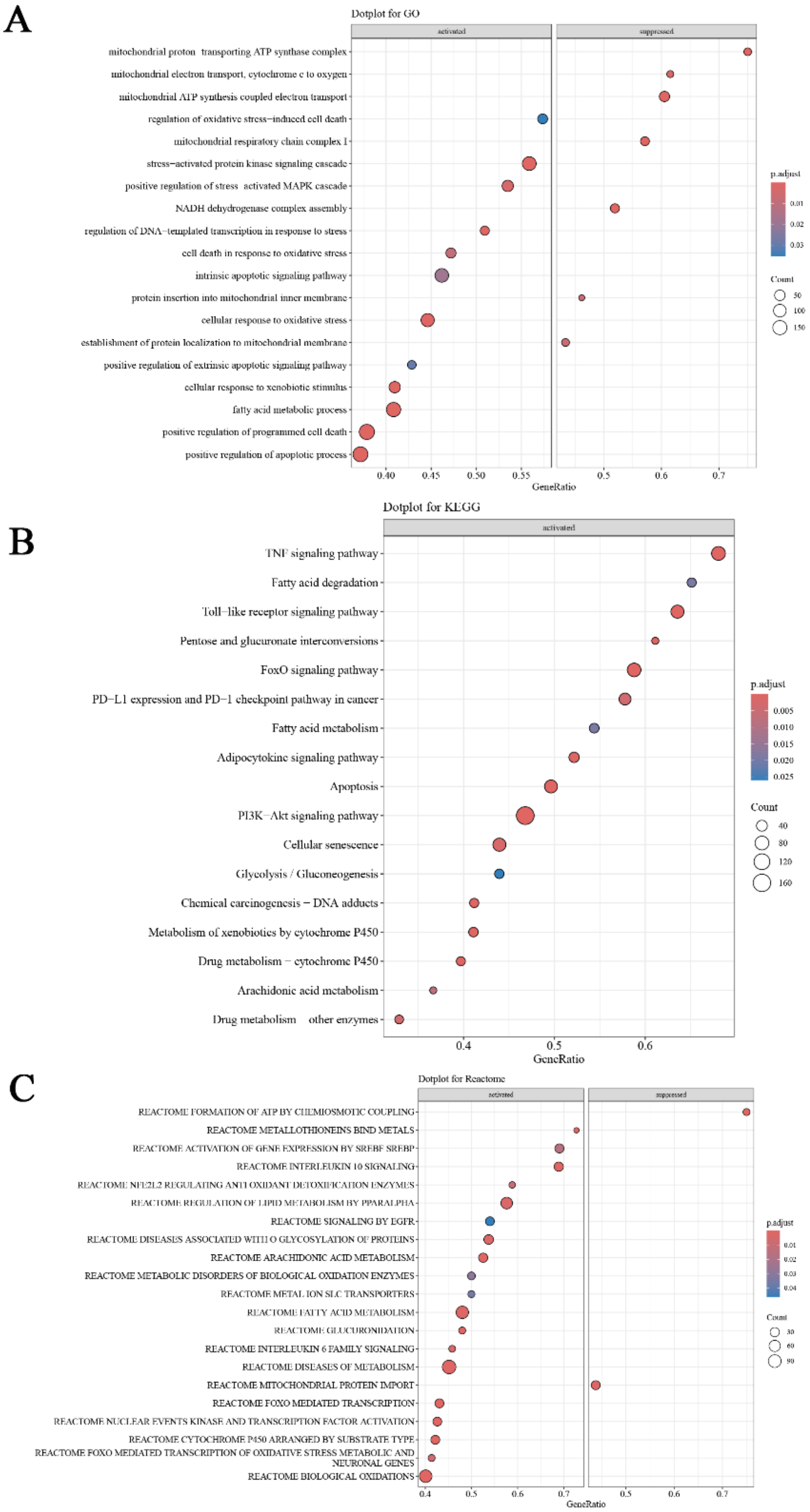

Supplementary Figure 2. Functional enrichment analysis of genes associated with ATF3 expression in TCGA. Panels display the most significantly enriched terms from (A) Gene Ontology (GO), (B) KEGG pathway, and (C) Reactome pathway analyses.

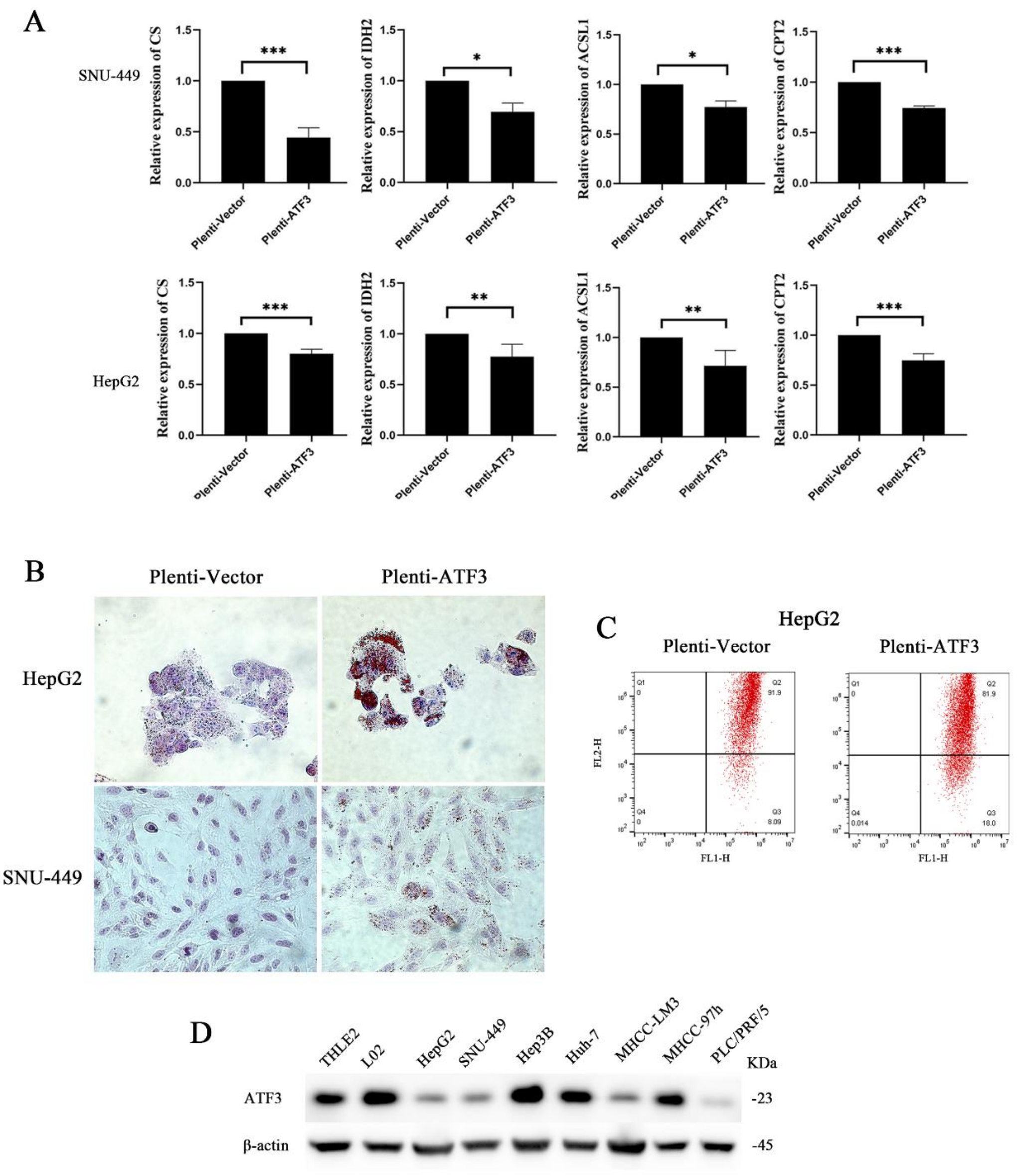

Supplementary Figure 3. Overexpression of ATF3 induces mitochondrial dysfunction and lipid accumulation in HCC cell lines. A. Overexpression of ATF3 in HCC cell lines resulted in downregulation of CS, IDH2, ACSL1, and CPT2 expression. B. Oil Red O staining showing the overexpression of ATF3 and accumulation of intracellular lipid droplets in liver cancer cell lines (HepG2 and SNU-449) at 400× magnification. C. Overexpression of ATF3 in liver cancer cell line (HepG2) leads to a decrease in mitochondrial membrane potential. D. Detection of ATF3 protein expression in normal liver cell lines and various liver cancer cell lines. $*p < 0.05$, $**p < 0.01$, $***p < 0.001$. CS: Citrate Synthase, IDH2: Isocitrate Dehydrogenase 2, ACSL1: Acyl-CoA Synthetase Long Chain Family Member 1, and CPT2: Carnitine Palmitoyl Transferase 2.

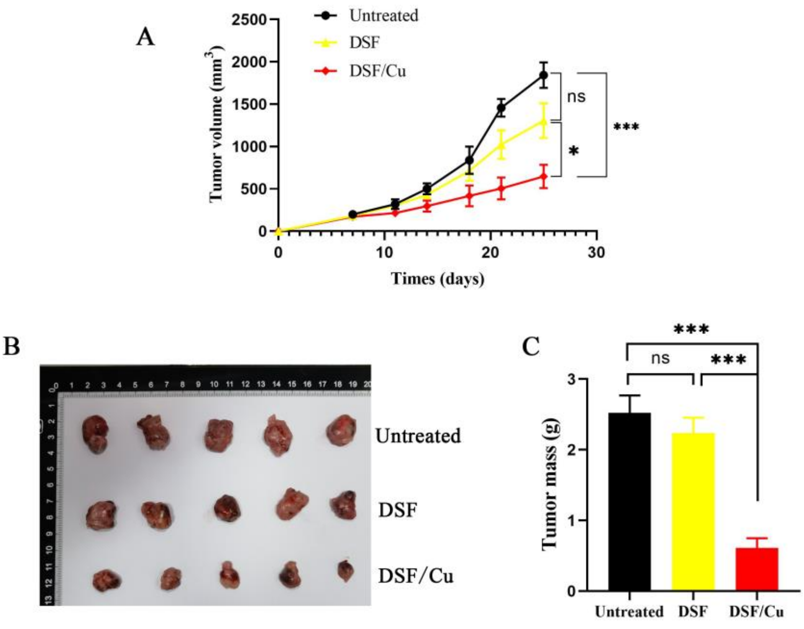

Supplementary Figure 4. DSF/Cu combination therapy potently inhibits tumor growth *in vivo*. A. Tumor growth curve showing the effect of DSF/Cu treatment on tumor volume over time. Compared to the single treatment or control groups, the DSF/Cu combination treatment group shows a significant reduction in tumor volume. B. Representative images of tumors from each treatment group. C. Tumor volume map comparing tumor size across treatment groups.

Supplementary Tables

Supplementary Table 1. Target genes and their corresponding qPCR primer sequences.

ACSL1-F	CTTATGGGCTTCGGAGCTTTT
ACSL1-R	CAAGTAGTGCGGATCTTCGTG
CPT2-F	CTGGAGCCAGAAGTGTTCCAC
CPT2-R	AGGCACAAAGCGTATGAGTCT
CS-F	TCCGACCCTTACCTGTCCTT
CS-R	ACTTCCTGATTTGCCAGTCC
OGDH-F	TTTGGTCTAGAAGGCTGCGA
OGDH-R	TCTGTTCCAGCTCCTTCCTG
β-actin-F	GCACCCAGCACAATGAAGAT
β-actin-R	ACATCTGCTGGAAGGTGGAC
ATF3-F	CCTCTGCGCTGGAATCAGTC
ATF3-R	TTCTTTCTCGTCGCCTCTTTTT

Supplementary Table 2. List of Antibodies for Western Blot Analysis.

	Product number	Brand
ATF3	EPR19488	Abcam
Bax	2772	Cell Signaling Technology (**CST**)
Bcl-2	3498	Cell Signaling Technology (**CST**)
Cleaved caspase 3	9664	Cell Signaling Technology (**CST**)
Cleaved caspase 9	9509	Cell Signaling Technology (**CST**)
Cytochrome c	ab13575	Abcam
β-actin	8H10D10	Cell Signaling Technology (CST)

Supplementary Table 3. ATF3 siRNA Sequences for RNAi Experiments.

	Sense（5'-3'）	Antisense（5'-3'）
ATF3-382	AGACCCCUCGGGGUGUCCAdTdT	UGGACACCCCGAGGGGUCUdGdU
ATF3-664	AAGAUGAGAGAAACCUCUUUAdTdT	UAAAGAGGUUUCUCUCAUCUUdCdT
